# Associations of cigarette card games engagement with smoking susceptibility and smoking behaviors among Chinese children and adolescents: Findings from the Zhejiang Childhood Behavior and Health Cohort study

**DOI:** 10.18332/tid/218781

**Published:** 2026-05-26

**Authors:** Lu Hua Yu, Xiao Qing Lu, Xiao Jing Yang, Zhu Yu, Wei Jiang, Yin Shu Pan, Li Ping Yang, Jun Hu, Yun Qi Guan, Chun Xiao Xu, Wei Yuan Yao, Li Xin Wang, Jie Ming Zhong, Meng Wang

**Affiliations:** 1 Department of Chronic and Non-communicable Disease Control and Prevention, Yiwu Center for Disease Control and Prevention, Jinhua, China; 2 Department of Nursing, First Affiliated Hospital of Zhejiang University School of Medicine, Hangzhou, China; 3 Department of Chronic and Non-communicable Disease Control and Prevention, Shangyu Center for Disease Control and Prevention, Shaoxing, China; 4 Department of Endocrine, Children's Hospital of Zhejiang University School of Medicine, Hangzhou, China; 5 Department of Chronic and Non-communicable Disease Control and Prevention, Haiyan Center for Disease Control and Prevention, Jiaxing, China; 6 Department of Chronic and Non-communicable Disease Control and Prevention, Yueqing Center for Disease Control and Prevention, Wenzhou, China; 7 Department of Chronic and Non-communicable Disease Control and Prevention, Nanxun Center for Disease Control and Prevention, Huzhou, China; 8 Department of Chronic and Non-communicable Disease Control and Prevention, Longyou Center for Disease Control and Prevention, Quzhou, China; 9 Department of Chronic and Non-communicable Disease Control and Prevention, Zhejiang Provincial Center for Disease Control and Prevention, Hangzhou, China

**Keywords:** cigarette packaging, smoking susceptibility, public health, electronic cigarette

## Abstract

**INTRODUCTION:**

Recently, cigarette card games have gained great popularity among Chinese schoolchildren. This study aimed to examine the associations of cigarette card game engagement with smoking susceptibility and smoking-related behaviors in students.

**METHODS:**

Using data from the Zhejiang Childhood Behavior and Health Cohort, 21526 children and adolescents aged 6–19 years were included, and relevant information on cigarette card games, smoking susceptibility, and several smoking behaviors was collected via self-reported questionnaires. Logistic regression models were used to explore the influence of cigarette card games on smoking susceptibility, as well as the smoking behaviors. Odds ratios (ORs) and their 95% confidence intervals (CIs) are reported.

**RESULTS:**

Among the 21526 participants, 861 (4.00%) reported engaging in the cigarette card games within the past 7 days. Never smoking students who engaged in cigarette card games exhibited greater odds of smoking susceptibility than those who did not engage (adjusted odds ratio, AOR=2.05; 95% CI: 1.70–2.46). Similarly, increased odds of ever cigarette smoking, ever and current e-cigarette use were also observed among those participating in such games, with AORs of 3.73 (95% CI: 2.83–4.90), 3.21 (95% CI: 2.31–4.46), and 6.87 (95% CI: 4.87–9.69), respectively.

**CONCLUSIONS:**

Findings of the present study confirmed the popularity of cigarette card games among Chinese students. They indicated that engagement in these games was significantly associated with increased odds of smoking susceptibility and various smoking-related behaviors. However, given the limitations of the study, these conclusions should be interpreted with caution.

## INTRODUCTION

The World Health Organization (WHO) has reported that tobacco causes the deaths of over 7 million individuals annually^1^. While it is encouraging to note a steady decline in tobacco use since 1990 among individuals aged ≥15 years^2^, the disease burden associated with tobacco remains substantial, with an uptrend due to population growth and morbidity and mortality attributable to tobacco^3^. Since most adult smokers began smoking during adolescence and tobacco is highly addictive^4,5^, tobacco use among the youth population has emerged as a critical public health challenge. The Global Youth Tobacco Survey (GYTS) confirmed that nearly 80% of adolescents began smoking cigarettes before the age of 13 years^6^, and 17.4% of adolescents aged 13–15 years smoked cigarettes on at least one day during the preceding 30 days^7^. To some extent, these findings have established youth tobacco use as a global public health priority and highlighted the urgent need to identify emerging determinants. Previous studies indicate that the smoking behavior of children and adolescents is influenced by a range of traditional socio-economic, environmental, and behavioral factors^8-10^. In contrast, the impact of emerging cigarette card games on smoking susceptibility and related behaviors has not been extensively investigated.

Cigarette cards are made from cigarette packs by removing the boxes and shaping the packs into rectangular shapes. In one form of the cigarette card game, children and adolescents would place the cards on the ground, take turns by holding one’s hands together and hitting the ground with their palms open. If a card or more are flipped over, the individual wins the cards^11^. Since mid-2023, cigarette card games have experienced an unexpected surge in popularity among Chinese students^12^. Although nationwide data are limited, a population-representative cross-sectional study conducted in Shenzhen City in 2023 revealed that at least 3.9% of schoolchildren have participated in the cigarette card games^13^. More importantly, some deep concerns arose from findings that card playing and collection could enhance children’s knowledge of cigarettes^14^. This increased awareness may further entice youth to experiment with smoking, thereby undermining primary efforts to prevent smoking initiation. To date, direct evidence quantifying the associations between engagement in cigarette card games and smoking susceptibility, as well as smoking behaviors, remains notably lacking.

Therefore, to address this knowledge gap, this study utilized data from the Zhejiang Childhood Behavior and Health Cohort (ZCBHC) to investigate the impact of participation in cigarette card games on smoking susceptibility and smoking-related behaviors among Chinese children and adolescents.

## METHODS

### Study design and participants

The ZCBHC study was initiated to prospectively investigate individual health behaviors and their subsequent association with health outcomes among school-aged children and adolescents in China. Comprehensive methodological details have been outlined in prior publications^15^. In brief, the survey of ZCBHC was conducted from September 2024 to January 2025, involving 21698 students (mean age=11.6 years; 46.8% girls) from primary schools (Grades 1–6), junior middle schools (Grades 7–9), and junior high schools (Grades 10–12) across six diverse regions of Zhejiang Province. Demographic characteristics and individual health behaviors of participants, including physical activity, dietary habits, cigarette smoking, alcohol consumption, sleep duration, and other related factors, were collected using a self-administered questionnaire based on the US Youth Risk Behavior Surveillance System (YRBSS) and the Global School-based Health Survey (GSHS). To ensure developmental appropriateness and address school requests, certain items related to suicidal and sexual behaviors were excluded from the primary-school version. Students in Grade 4 and above completed an anonymous questionnaire independently in the classroom, after which the researchers collected the questionnaires immediately. For pupils in Grades 1–3, given their limited cognitive abilities, the questionnaire was sent home for completion with the assistance of parents or guardians. Anthropometric data, including body weight, standing height, waist circumference, and blood pressure, were collected during the annual school physical examination conducted by certified health workers, with administrative support from the Ministry of Education. To ensure voluntary participation, parents and guardians received a written opt-out information letter before data collection, which outlined the study objectives, data collection methods, and consent for data use and publication. All researchers completed mandatory privacy-protection training, and all procedures adhered to the Declaration of Helsinki. Furthermore, this study received approval from the ethics committee of the Zhejiang Provincial Center for Disease Control and Prevention (approval No. 2024-027-01).

### Study variables definition

Self-reported smoking susceptibility and smoking-related behaviors were the main outcomes of interest. On the questionnaire, a never smoking student was defined as being susceptible to cigarette smoking based on their response options (definitely yes, probably yes, probably not, and definitely not) to the three following questions: 1) ‘Do you think you will smoke a cigarette soon?’; 2) ‘Do you think you will smoke a cigarette in the next year?’; and 3) If one of your best friends offered you a cigarette, would you smoke it?’. A student who responded ‘definitely not’ to all three questions was defined as not susceptible, and any other response to the three questions was defined as being susceptible to cigarette smoking. The ever cigarette smoking and e-cigarette use behaviors among children and adolescents were identified if they answered yes to the questions, respectively: ‘Have you ever tried cigarette smoking, even one or two puffs?’ and ‘Have you ever tried e-cigarette, even one or two puffs?’. The following question assessed current e-cigarette use: ‘During the past 30 days, on how many days did you use an e-cigarette?’ (0, 1–2, 3–5, 6–9, 10–19, 20–29, or 30 days)’. Children and adolescents were considered current users if they answered that they had used an e-cigarette at least 1 day during the past 30 days. The engagement in cigarette card games was the study exposure variable and assessed by the question: ‘During the past 7 days, how many days did you play the cigarette card games? (0, 1, 2, 3, 4, 5, 6, or 7 days)’. Participants who answered at least 1 day were considered to have engaged in the cigarette card games.

### Other covariates

The following potential confounders were simultaneously adjusted for in the analysis: sex (boys, girls), age group (6–10, 11–15, 16–20 years), school location (rural, urban), school level (primary, middle, high), only-child status (yes, no), smokers in family members (yes, no), and secondhand smoke exposure (yes, no).

### Statistical analysis

Descriptive statistics were used to estimate cigarette card game engagement, and the prevalence in participants with different characteristics was compared using the chi-squared test and the linear-by-linear association chi-squared test. A p<0.05 was considered to be statistically significant. To explore the possible associations of cigarette card game engagement with smoking susceptibility and related behaviors among children and adolescents, multivariable logistic regression was performed in three sequential models, reporting adjusted odds ratios (AORs) with 95% confidence intervals (CIs). Model 1 adjusted for the sociodemographic characteristics of age and sex. Model 2 further adjusted for the location of school, school level, and only-child status. Model 3 adjusted as for Model 2 plus smokers in family members and secondhand smoke exposure. Besides, based on Model 3, subgroup analyses were conducted to test whether the associations of cigarette card game engagement with smoking susceptibility and smoking-related behaviors were affected and varied by the main sociodemographic characteristics, including sex, age, school location, and school level. Finally, to evaluate the robustness of the estimates, we conducted the sensitivity analyses by excluding the participants who were exposed to secondhand smoke and from high schools with extremely low prevalence of playing cigarette card games. All analyses were performed using the SAS statistical package (version 9.4, SAS Institute, Inc., Cary, NC, USA).

## RESULTS

### The characteristics of participants

Among the included participants (n=21526) with ages ranging from 6 to 19 years, there were 861 (4.00%) children and adolescents who had engaged in the cigarette card games in the past 7 days. The characteristics of participants according to their engagement in cigarette card games are shown in Table 1. The participants engaging in cigarette card games tended to be boys, younger, from urban schools, primary schools, and having smokers in family members and been exposed to secondhand smoke (all p<0.001).

**Table 1:** Characteristics of ZCBHC participants according to engagement in cigarette card games in the past 7 days (N=21526)

*Characteristics*	*Playing cigarette card games ^a^*		*p*
	*Yes* *n (%)*	*No* *n (%)*	
**Total**	861 (4.00)	20665 (96.00)	
**Sex**			<0.001
Boys	663 (5.79)	10788 (94.21)	
Girls	198 (1.97)	9877 (98.03)	
**Age** (years)^b^			<0.001
6–10	446 (5.90)	7113 (94.10)	
11–15	410 (3.30)	12013 (96.70)	
16–20	5 (0.32)	1539 (99.68)	
**Location of school**			<0.001
Rural	513 (3.49)	14170 (96.51)	
Urban	348 (5.09)	6495 (94.91)	
**School level** ^b^			<0.001
Primary school	521 (5.92)	8282 (94.08)	
Middle school	332 (3.21)	10006 (96.79)	
High school	8 (0.34)	2377 (99.66)	
**Only child**			0.105
Yes	227 (3.67)	5963 (96.33)	
No	634 (4.15)	14656 (95.85)	
**Smokers in family**			<0.001
Yes	618 (4.69)	12553 (95.31)	
No	241 (2.90)	8080 (97.10)	
**Secondhand smoke exposure**			<0.001
Yes	742 (4.35)	16296 (95.65)	
No	112 (2.63)	4141 (97.37)	

ZCBHC: Zhejiang Childhood Behavior and Health Cohort.

a The cigarette card games engagement data of 172 participants were missing.

b Difference between these groups was compared using linear-by-linear association χ^2^ test.

### Associations of cigarette card game engagement with smoking susceptibility and smoking-related behaviors

Table 2 showed the associations of cigarette card game engagement with smoking susceptibility and related behaviors. After adjustment for the potential confounding factors, the never smoking children and adolescents who engaged in cigarette card games in the past 7 days had greater odds of susceptibility to cigarette smoking than those who never did (AOR=2.05; 95% CI: 1.70–2.46). For smoking-related behaviors, similarly, children and adolescents who engaged in cigarette card games had increased odds of ever cigarette smoking, ever and current use of e-cigarette than their counterparts, with the AORs being 3.73 (95% CI: 2.83–4.90), 3.21 (95% CI: 2.31–4.46), and 6.87 (95% CI: 4.87–9.69), respectively.

**Table 2:** Adjusted ORs (95% CIs) of susceptibility to cigarette smoking and smoking-related behaviors by engagement in cigarette card games

*Outcomes*	*Exposure/ total*	*Model 1* *AOR (95% CI)*	*Model 2* *AOR (95% CI)*	*Model 3* *AOR (95% CI)*
**Susceptibility to cigarette smoking**				
No (ref.)	612/18199	1.00	1.00	1.00
Yes	167/2603	2.09 (1.75–2.50)	2.14 (1.78–2.56)	2.05 (1.70–2.46)
**Ever cigarette smoking**				
No (ref.)	785/20880	1.00	1.00	1.00
Yes	73/616	4.18 (3.20–5.44)	4.05 (3.09–5.31)	3.73 (2.83–4.90)
**Ever e-cigarette use**				
No (ref.)	801/20960	1.00	1.00	1.00
Yes	48/459	3.68 (2.68–5.06)	3.52 (2.55–4.86)	3.21 (2.31–4.46)
**Current e-cigarette use**				
No (ref.)	812/21254	1.00	1.00	1.00
Yes	48/239	7.28 (5.21–10.19)	6.80 (4.84–9.56)	6.87 (4.87–9.69)

AOR: adjusted odds ratio. Model 1 adjusted for age and sex. Model 2 adjusted as for Model 1 plus location of school, school level, and only child. Model 3 adjusted as for Model 2 plus smokers in family members and secondhand smoke exposure.

Table 3 shows that the association of cigarette card games engagement was more pronounced with current e-cigarette use in students from rural schools and was more pronounced with ever smoking and ever e-cigarette use in students from primary schools. The interaction test and heterogeneity test showed consistent results for these corresponding outcomes (all p≤0.05).

**Table 3:** Fully adjusted ORs (95% CIs) of susceptibility to cigarette smoking and smoking-related behaviors by engagement in cigarette card games within subgroups

*Subgroup*	*Exposure/* *total*	*Susceptibility to* *cigarette smoking,* *exposure/outcome* *AOR (95% CI)*	*Ever smoking,* *exposure/outcome* *AOR (95% CI)*	*Ever e-cigarette use,* *exposure/outcome* *AOR (95% CI)*	*Current-cigarette use,* *exposure/outcome* *AOR (95% CI)*
**Sex**					
Boys	663/11451	132/1604; 1.93 (1.57–2.38)	57/413; 3.64 (2.66–4.99)	38/285; 3.38 (2.31–4.93)	36/127; 7.42 (4.88–11.27)
Girls	198/10075	35/1010; 2.44 (1.66–3.59)	16/207; 4.67 (2.68–8.15)	10/177; 3.24 (1.65–6.37)	12/112; 6.28 (3.34–11.79)
p for interaction^a^		0.35	0.24	0.83	0.77
p for heterogeneity^b^		0.29	0.45	0.92	0.67
**Age (years)**					
6–10	446/7559	81/627; 2.40 (1.84–3.13)	23/67; 6.91 (4.02–11.89)	16/44; 7.33 (3.81–14.12)	18/50; 9.33 (5.02–17.32)
≥11	416/13967	340/1987; 1.78 (1.38–2.29)	50/553; 3.06 (2.12–4.24)	32/418; 2.50 (1.69–3.70)	38/189; 5.84 (3.82–8.92)
p for interaction^a^		0.95	0.15	0.05	0.28
p for heterogeneity^b^		0.11	0.01	0.01	0.22
**Location of school**					
Rural	513/14683	87/1712; 1.88 (1.47–2.40)	35/319; 4.83 (3.26–7.15)	22/235; 4.54 (2.80–7.37)	17/130; 10.88 (6.96–16.99)
Urban	348/6843	80/902; 2.30 (1.74–3.03)	38/301; 3.00 (2.05–4.38)	26/227; 2.43 (1.56–3.78)	31/109; 3.99 (2.30–6.90)
p for interaction^a^		0.06	0.09	0.21	0.002
p for heterogeneity^b^		0.29	0.09	0.06	0.01
**School level**					
Primary school	521/8803	91/720; 2.34 (1.82–3.00)	32/91; 7.28 (4.57–11.59)	21/61; 6.77 (3.85–11.88)	22/69; 8.22 (4.77–14.16)
Middle and high school	340/12723	76/1894; 1.73 (1.32–2.26)	41/529; 2.47 (1.74–3.51)	27/401; 2.12 (1.39–3.24)	26/170; 6.46 (4.11–10.16)
p for interaction^a^		0.23	0.01	0.02	0.68
p for heterogeneity^b^		0.11	<0.001	0.001	0.51

AOR: adjusted odds ratio.

a The p for interaction was calculated based on Model 3, which adjusted for sociodemographic characteristics of participants.

b The p for heterogeneity within subgroups was calculated using Cochran’s Q test.

In the sensitivity analyses, the associations of cigarette card game engagement with smoking susceptibility and related behaviors did not change substantially after excluding participants with secondhand smoke exposure and those from high school, respectively (Table 4).

**Table 4:** Sensitivity analyses: Adjusted odds ratios (95% CIs) of susceptibility to cigarette smoking and smoking-related behaviors by engagement in cigarette card games

*Outcomes*	*Cases/ total*	*Model 1* *AOR (95% CI)*	*Model 2* *AOR (95% CI)*	*Model 3* *AOR (95% CI)*
**Excluding participants with secondhand smoke exposure**				
**Susceptibility to cigarette smoking**				
No (ref.)	84/3841	1.00	1.00	1.00
Yes	21/349	2.83 (1.72–4.65)	2.87 (1.75–4.73)	2.85 (1.73–4.70)
**Ever cigarette smoking**				
No (ref.)	106/4207	1.00	1.00	1.00
Yes	5/43	4.82 (1.82–12.71)	4.55 (1.72–12.06)	4.29 (1.61–11.43)
**Ever e-cigarette use**				
No (ref.)	106/4197	1.00	1.00	1.00
Yes	4/36	4.73 (1.61–13.95)	4.86 (1.63–14.45)	4.44 (1.48–13.36)
**Current e-cigarette use**				
No (ref.)	97/4189	1.00	1.00	1.00
Yes	15/60	14.49 (7.65–27.42)	14.38 (7.54–27.44)	14.46 (7.55–27.69)
**Excluding participants from high school**				
**Susceptibility to cigarette smoking**				
No (ref.)	607/16324	1.00	1.00	1.00
Yes	167/2230	2.13 (1.78–2.55)	2.18 (1.82–2.61)	2.08 (1.73–2.50)
**Ever cigarette smoking**				
No (ref.)	780/18625	1.00	1.00	1.00
Yes	70/486	4.33 (3.30–5.68)	4.04 (3.06–5.32)	3.68 (2.78–4.87)
**Ever e-cigarette use**				
No (ref.)	795/18675	1.00	1.00	1.00
Yes	46/364	3.85 (2.78–5.34)	3.52 (2.53–4.90)	3.19 (2.28–4.47)
**Current e-cigarette use**				
No (ref.)	806/18889	1.00	1.00	1.00
Yes	46/222	6.92 (4.91–9.71)	6.52 (4.61–9.21)	6.54 (4.60–9.29)

Model 1 adjusted for age and sex. Model 2 adjusted as for Model 1 plus location of school, school level, and only child. Model 3 adjusted as for Model 2 plus smokers in family members and secondhand smoke exposure (except in the excluding participants with secondhand smoke exposure).

## DISCUSSION

Involving 21526 Chinese school-attending children and adolescents aged 6–19 years, the present study revealed that approximately 4% of participants engaged in cigarette card games during the 7 days preceding the survey. Moreover, after adjusting for potential confounders, this study provided the novel population-level evidence that participation in cigarette card games was significantly associated with a two-fold increase in smoking susceptibility and a three- to six-fold increase in various smoking-related behaviors, such as ever smoking and ever/current e-cigarette use among children and adolescents. These associations were generally consistent across most subgroups defined by sociodemographic characteristics and remained robust in sensitivity analyses.

Using data from the ZCBHC study conducted in 2024, our findings indicated that the overall prevalence of engagement in cigarette card games among all included students was 4.00%. This prevalence was 5.92% for primary school students and 3.21% for middle school students. Although the cohort study was not specifically designed to characterize the behavioral and health-risk profiles of participants across research areas utilizing a non-probability sampling method, our overall estimate closely aligned with the reported rate of cigarette card game participation (3.9%) among children in Shenzhen in late 2023^13^. These preliminary data collected in 2023 and 2024 strongly confirm the popularity of cigarette card games among Chinese teenagers since their emergence. Besides, despite the absence of nationwide data, the reported figures from relatively developed regions of China clearly confirmed that market supervision and tobacco monopoly departments have had limited success in curbing the spread of cigarette cards, especially from online sources^16^. Furthermore, our analysis revealed that participation in cigarette card games was significantly associated with increased odds of smoking susceptibility and various smoking-related behaviors. To some extent, these findings would support earlier concerns that such games may be associated with smoking experimentation among youth, ultimately undermining anti-smoking efforts within this population^17^. The mechanisms underlying the sudden popularity of cigarette card games in China, as well as their significant association with smoking susceptibility and related behaviors, remain complex and underexplored. Nonetheless, a recent qualitative study found that appealing cigarette packaging motivated children’s participation in cigarette card games, which subsequently normalized smoking by fostering brand familiarity and facilitating social interactions among peers^14^. Indeed, Chinese cigarette packs often feature cultural appeals^18^, with a significant gap in implementing effective packaging regulations recommended by the WHO Framework Convention on Tobacco Control (FCTC)^19^. Consequently, the absence of plain packaging has created an opportunity for children to participate in cigarette card games, thereby acquiring these visually striking packs. Furthermore, the design of cigarette packaging serves as a marketing promotion strategy, allowing children to become familiar with brands, logos, and prices through gameplay^14^. A previous study indicated that children are universally exposed to tobacco promotion prior to the onset of tobacco use, which is causally linked to the initiation of tobacco use^20^. Another review study found that non-smoking adolescents who were more aware of or receptive to tobacco advertising were more likely to have experimented with cigarettes or to have become smokers at follow-up^21^. A cross-sectional observational study of high school students in Scotland found that greater recognition of cigarette brands was linked to an increased intention to use e-cigarettes and a higher likelihood of prior use^22^.

### Limitations

This study has several limitations. First, due to the cross-sectional design of the analysis, the possible causal relationships between engagement in cigarette card games and smoking susceptibility, as well as various smoking-related behaviors, cannot be definitively established. Second, the participants’ information for this survey was gathered through self-administered questionnaires, and the validity of the responses was not assessed. Consequently, we hypothesized that the data utilized in this analysis, including the evaluation of cigarette card game playing, as well as the susceptibility to cigarette smoking, ever smoking, and ever/current e-cigarette use, may be influenced by reporting bias. Besides, the time windows used for cigarette card game playing (in 7 days), as well as smoking susceptibility (in 12 months) and smoking behaviors (lifetime or in 30 days) assessment, were not relatively consistent in the questionnaire, which may bring about temporal bias in the analysis and overestimate these associations. Third, because the sample of participants was drawn from schools only in Zhejiang Province, caution is warranted in interpreting and generalizing these findings to the wider population of Chinese children and adolescents, as their fundamental characteristics may differ, and some individuals may not be enrolled in school.

## CONCLUSIONS

This study confirmed the widespread engagement of Chinese students in cigarette card games in Zhejiang Province and indicated that participation in these games is significantly associated with increased susceptibility to smoking and smoking-related behaviors. Although there are some limitations of this study, these noteworthy findings carry important implications for both research and practice. For instance, additional nationwide studies are necessary to monitor the prevalence trends of cigarette card games among Chinese children and adolescents. These studies should also aim to identify the causal relationships between these games and youth smoking initiation, as well as the underlying mechanisms involved. In light of continuously updated evidence, policy-makers should promote the rigorous implementation of FCTC regulations on the packaging of tobacco products and the development of regulations on the circulation of cigarette cards. Besides, school health professionals should acknowledge and integrate the adverse effects of cigarette card games into educational programs for both students and parents.

## Data Availability

The data supporting this research are available from the authors on reasonable request.
